# Conformational Change Induced by Putidaredoxin Binding to Ferrous CO-ligated Cytochrome P450cam Characterized by 2D IR Spectroscopy

**DOI:** 10.3389/fmolb.2018.00094

**Published:** 2018-11-13

**Authors:** Sashary Ramos, Edward J. Basom, Megan C. Thielges

**Affiliations:** Department of Chemistry, Indiana University, Bloomington, IN, United States

**Keywords:** cytochrome P450, putidaredoxin, protein dynamics, energy landscape, infrared spectroscopy, 2D IR spectroscopy

## Abstract

The importance of conformational dynamics to protein function is now well-appreciated. An outstanding question is whether they are involved in the effector role played by putidaredoxin (Pdx) in its reduction of the O_2_ complex of cytochrome P450cam (P450cam), an archetypical member of the cytochrome P450 superfamily. Recent studies have reported that binding of Pdx induces a conformational change from a closed to an open state of ferric P450cam, but a similar conformational change does not appear to occur for the ferrous, CO-ligated enzyme. To better understand the effector role of Pdx when binding the ferrous, CO-ligated P450cam, we applied 2D IR spectroscopy to compare the conformations and dynamics of the wild-type (wt) enzyme in the absence and presence of Pdx, as well as of L358P P450cam (L358P), which has served as a putative model for the Pdx complex. The CO vibrations of the Pdx complex and L358P report population of two conformational states in which the CO experiences distinct environments. The dynamics among the CO frequencies indicate that the energy landscape of substates within one conformation are reflective of the closed state of P450cam, and for the other conformation, differ from the free wt enzyme, but are equivalent between the Pdx complex and L358P. The two states co-populated by the Pdx complex are postulated to reflect a loosely bound encounter complex and a more tightly bound state, as is commonly observed for the dynamic complexes of redox partners. Significantly, this study shows that the binding of Pdx to ferrous, CO-ligated P450cam does perturb the conformational ensemble in a way that might underlie the effector role of Pdx.

## Introduction

Protein function requires dynamics, the population of and interconversion among multiple conformations and substates of a hierarchical energy landscape. They have been implicated in the activity of cytochrome P450s (CYPs), a superfamily of heme-thiolate enzymes that catalyze oxygen insertion from molecular dioxygen in diverse substrates for a wide range of biosynthetic and metabolic processes (Sigel et al., 2007; Ortiz de Montellano, 2015). For example, crystal structures for a number of CYPs show conformational adaptation for substrate recognition (Williams et al., 2000; Scott et al., 2003; Wester et al., 2003). The most comprehensively studied CYP, P450cam from *Pseudomonas putida*, undergoes a conformational change from an “open” to a “closed” state upon binding its substrate, camphor (Lee et al., 2010; Asciutto et al., 2011). Whether conformational dynamics play a role in the catalytic cycle beyond substrate recognition is currently a question of high interest (Hollingsworth and Poulos, 2015; Liou et al., 2016, 2017; Batabyal et al., 2017).

**Figure 1:**
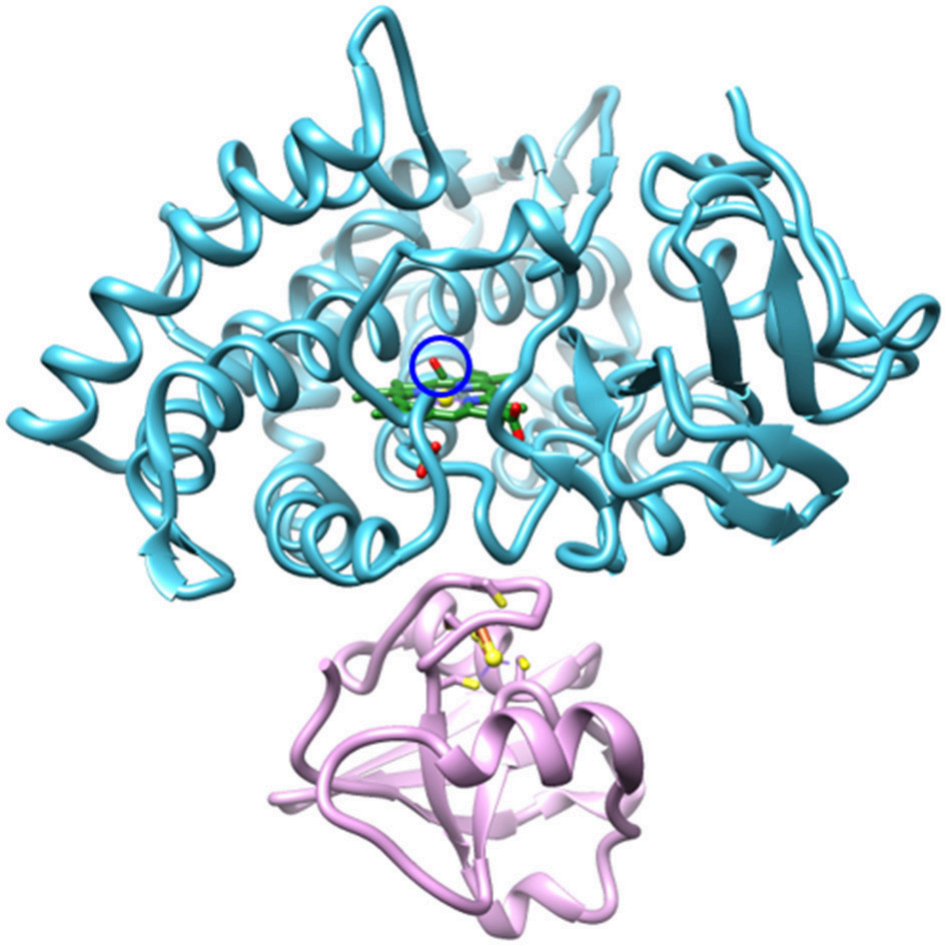
Structural model (PDB ID: 4JWU) of the complex of P450cam (blue) and Pdx (pink). The CO IR probe is circled in blue, the heme is shown in green, and the Fe atoms are shown as yellow spheres. Image generated with UCSF Chimera (Pettersen et al., 2004).

CYPs generally undergo a common catalytic cycle (Denisov et al., 2005). Briefly, the binding of substrate to the ferric enzyme displaces a heme-ligated water molecule, leading to conversion of the heme from low to high spin and an increase in redox potential. This promotes a one-electron reduction, followed by binding of O_2_. A second one-electron reduction and uptake of two protons generates a highly reactive oxo-ferryl porphyrin radical species, compound I, which is responsible for substrate oxidation. The reduction of CYPs is typically nonspecific and can be mediated by a range of protein and small molecule reductants. However, P450cam is an outlier as the second reduction step has a strict requirement for the native redox partner, the iron-sulfur protein putidaredoxin (Pdx) (Figure 1; Tyson et al., 1972).

A combination of spectroscopic, mutational, thermodynamic, and kinetic binding studies helped to generate the first models of the Pdx complex with P450cam (Sligar et al., 1974; Hintz and Peterson, 1981; Koga et al., 1993; Pochapsky et al., 1994, 1996; Holden et al., 1997; Unno et al., 1997; Kuznetsov et al., 2005, 2006; Zhang et al., 2008; Sevrioukova et al., 2010). A fortuitous observation was made that the mutation, L358P, which removes an interaction of the amide backbone with the thiolate of the cysteine heme ligand, engenders similar spectral changes as binding to Pdx and enables catalysis of camphor hydroxylation with small molecule reductants (Yoshioka et al., 2000, 2002; Tosha et al., 2004). The structure of the L358P P450cam (L358P) subsequently became a potential model for P450cam in the Pdx complex (Nagano et al., 2004; Tosha et al., 2004). Recently, crystal structures have been solved for the Pdx complex (Hiruma et al., 2013; Tripathi et al., 2013). These show a conformational change in P450cam from the closed state of the complex with the camphor substrate to the open state, previously observed in absence of substrate, which reinvigorated interest in the mechanism underlying the effector role of Pdx. However, whether the P450cam adopts an open state in the Pdx complex came into question by an NMR study that indicated a closed state; although, the cyanide complex was under investigation (Skinner et al., 2015). Subsequent double electron-electron resonance (DEER), mutational, and molecular dynamics studies of the unligated enzyme support an open, or partially open, intermediate state (Hollingsworth and Poulos, 2015; Liou et al., 2016, 2017; Batabyal et al., 2017). Complicating the picture is the apparent dependence of the conformational dynamics on the oxidation state of P450cam (Myers et al., 2013; Liou et al., 2016). However, a consensus is forming that Pdx binding indeed promotes conversion to the open state for the ferric enzyme, whereas the ferrous, CO-ligated enzyme appears to remain in the closed state.

The question remains whether a conformational change underlies the effector role of Pdx that is critical to the second reduction step involving the O_2_ complex of P450cam. Kinetic studies suggest that the first and second reduction steps involve either different complexes between Pdx and P450cam or different electron transfer pathways (Kuznetsov et al., 2006). Although Pdx does not appear to induce the open state of ferrous CO-ligated P450cam, Raman, infrared (IR), and NMR studies found evidence for spectral perturbation upon Pdx binding (Unno et al., 2002; Nagano et al., 2003; Pochapsky et al., 2003; Tosha et al., 2003; Wei et al., 2005; Asciutto et al., 2009), suggesting that the structure of P450cam could be affected in a way that is connected to the effector role of Pdx in the second reduction step of the catalytic cycle. Toward elucidating the impact of Pdx binding, we applied two-dimensional (2D) IR spectroscopy to the CO-ligated camphor complex of P450cam in the presence and absence of Pdx (subsequently referred to as the Pdx complex and the free wt, respectively), as well as of L358P, the purported model for the P450cam structure in the Pdx complex. The CO vibration served as a reporter of its environment, including the number of distinct environments and the dynamics in each, providing insight into the number of populated conformations and the nature of the energy landscape within them. The time-dependent 2D IR data rigorously demonstrate that the Pdx complex and L358P co-populate two distinct states. For the Pdx complex, the closed state found for the free wt enzyme is the minor species, while a second conformation, which could underlie the effector function, is the dominantly populated state.

## Materials and methods

### Preparation ofup450cam

P450cam was recombinantly expressed with plasmid pDNC334A, and Pdx was expressed with plasmid pKM36 (kindly provided by Thomas Pochapsky, Brandeis University) (Lyons et al., 1996; OuYang et al., 2006). The mutation L358P was introduced into the P450cam gene sequence using site-directed mutagenesis (Stratagene, Agilent) and confirmed by sequencing. Expression and purification of the proteins was performed as previously described with some modifications (see Supplementary Material **Expression and Purification of Protein**; Basom et al., 2015; Liou et al., 2016). Purified proteins were dialyzed into 100 mM potassium phosphate, pH 7, 50 mM KCl, 5 mM *d*-camphor and spin-concentrated to 3–4 mM. The concentrated Pdx was reduced with 10 equivalents of dithiothreitol and stored under Ar_(g)_. To form the CO-ligated state of P450cam, wt or L358P was placed in a sealed tube and the headspace was gently purged with Ar_(g)_ for 10 min, then with CO_(g)_ for an additional 10 min. P450cam was reduced with 15 equivalents of sodium dithionite, and the headspace again was purged with CO_(g)_. To generate the Pdx complex, the reduced Pdx was added to the reduced P450cam to final concentrations of 1.6 and 1 mM, respectively.

### IR spectroscopy

The samples were loaded between 2 mm CaF_2_ windows separated by a 76 μm Teflon spacer. Visible spectra (Agilent Cary 300 Spectrometer) and FT IR spectra were collected of all samples to confirm binding of CO or Pdx and absence of the inactive P420 state (Supplementary Figure 1). All FT IR spectra were collected at 2 cm^−1^ resolution with an Agilent Cary 670 FT IR spectrometer equipped with a liquid N_2_-cooled MCT detector. Absorption spectra were generated from transmission spectra of the CO-ligated proteins and reference transmission spectra of the unligated proteins.

2D IR experiments were performed as previously described with some modifications (Park et al., 2007; Basom et al., 2015; Kramer et al., 2016). Briefly, a mid-IR beam (~20 μJ, 150 fs pulses, 1 kHz, centered at 1,920 cm^−1^) was split into four beams. Three were separately delayed and focused into the sample in a BOXCARS geometry. To generate a 2D IR spectrum, the time between the first two pulses, τ, was scanned while the time interval between the second and third pulses, the waiting time (*T*_*w*_), was fixed. The fourth beam, the local oscillator (LO), was overlapped with the third order signal emitted by the sample in the phase-matched direction for heterodyned detection. The combined beams were frequency-dispersed by a spectrograph onto a 32-element MCT array detector. Frequency-resolved detection directly generated the ω_3_ (vertical, probe) axis of the 2D spectrum, whereas Fourier transformation of the interferograms measured along τ generated the ω_1_ (horizontal, pump) axis. Data were collected using either stationary frame or quasi-rotating frame (QRF) implementations, the latter of which was introduced during the course of the study to reduce data acquisition time (Kramer et al., 2016). The data for the wt in the absence and presence of Pdx were collected using stationary frame, while that for L358P was collected using QRF (see Supplementary Material 2D **IR and Pump Probe Spectroscopy**). A QRF scan of the free wt was collected and compared to stationary frame and found to be identical within error (Supplementary Figure 5). Each sample was also characterized using pump probe spectroscopy (see Supplementary Material **2D IR and Pump Probe Spectroscopy**, Supplementary Figure 4). All experiments were performed in triplicate. Experiments were performed in a N_2_(g)-purged enclosure; however, bands associated with water vapor (e.g., at 1,918, 1,923, and 1,942 cm^−1^) can be seen in the 2D spectra of the Pdx complex taken at the longer *T*_w_ times, due to the 3- to 4-fold lower concentration of P450cam in this sample and the weaker signals for CO at longer *T*_w_ times resulting from more complete vibrational relaxation.

### Data analysis

Center line slope (CLS) analysis of the 2D lineshapes in combination with fitting to the linear IR spectra were used to determine the frequency-frequency correlation function (FFCF) (Kwak et al., 2007), a quantity which describes the dynamics of the protein environment coupled to the CO vibration. Briefly, the frequency of maximum absorbance along the ω_1_ axis from 1D slices of the 2D IR spectra at each ω_3_ (the center line data) were plotted, and the slope of the center line data were determined as a function of *T*_w_ to generate the CLS decay, which approximates the inhomogeneous part of the normalized FFCF. The FFCFs were analyzed according to the Kubo model using the equation (Kubo, 1969).

$$FFCF=\frac{\delta (t)}{T_{2}^{*}+2T_{1}}+\Delta_{1}^{2}e^{-t/\tau_{1}}+\Delta_{s}^{2}$$

The latter two terms described the dynamics among the inhomogeneous distribution of frequencies. The inhomogeneous dynamics were separated into two timescales, where Δ_1_ reflects the part of the frequency distribution sampled on the timescale τ_1_, and the static term Δ_*s*_, reflects the part of the frequency distribution sampled more slowly than the experimental time window. The first term accounted for the homogeneous contribution to the FFCF. *T*_1_ is the vibrational lifetime, which was measured by pump probe spectroscopy. The pure dephasing time, describes very fast fluctuations in the motionally narrowed limit where the frequency amplitude and timescale cannot be separated (Δτ≪1). The homogeneous dynamics lead to a Lorentzian contribution to the line shape, Γ∗=1/πT∗2.

The 2D spectra for the Pdx complex and L358P were further analyzed to account for multiple component bands. The FFCF of an unknown component can be extracted when the FFCF of one component and the relative populations of the associated states are known (Fenn and Fayer, 2011). Time-dependent fractional contributions of the two components were determined from the relative areas of the associated bands from band-fitting the linear and 2D diagonal spectra and the measured vibrational lifetimes. The center frequencies along the ω_1_ axis from 1D slices of the 2D IR spectra at each ω_3_ (the center line data) were extracted from the spectra for each variant. The average central line data from the three sets of 2D data were used in subsequent analysis. The center line data obtained from the 2D IR spectra of the Pdx complex and L358P were fit to two components, with the center line data for the component at higher frequency fixed to that obtained for the free wt enzyme. The FFCF of the second component was then determined from *T*_w_-dependent slopes of the center line data (see Supplementary Material **Two-component CLS Determination** for additional details).

## Results

**Figure 2:**
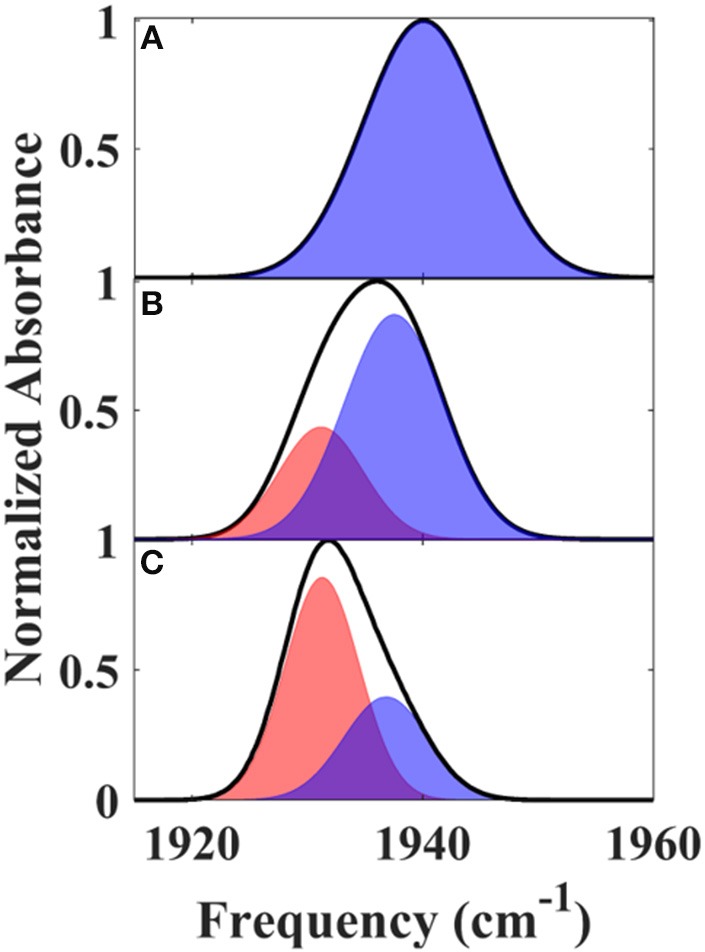
Linear FT IR spectra of heme-bound CO for **(A)** free wt, **(B)** L358P, and **(C)** the Pdx complex. Gaussian fits to the spectra are shown as shaded bands.

The reduced, CO-bound camphor complex of wt P450cam in the absence and presence of Pdx and of L358P first were characterized by linear spectroscopy (Figure 2). For the free wt, a single Gaussian absorption band was found at 1939.4 cm^−1^, as observed previously by our group and others (O'Keefe et al., 1978; Jung et al., 2002; Basom et al., 2015). In comparison to the free wt, the maxima of the spectra for the Pdx complex and L358P were lower in frequency by ~8 and 4 cm^−1^, respectively, as reported previously (Yoshioka et al., 2000; Nagano et al., 2003). However, closer inspection revealed asymmetry in the lineshapes for the Pdx complex and L358P, and second derivative spectra indicated the presence of multiple distinct bands (Supplementary Figure 3, Supplementary Table 1). Modeling the spectra as a sum of two Gaussian bands yielded a significantly better fit for both samples (Supplementary Figure 2; Table 1). The frequency of one band was similar to that found for the single band of free wt, differing only by ~2 cm^−1^, and the frequency of the other band was lower and the same for both the Pdx complex and L358P (1931.2 cm^−1^; Figure 2; Table 1). For the Pdx complex, the relative integrated absorbance of the band at lower frequency was greater than the one at higher frequency; while for L358P, the opposite was the case.

**Table 1:** Absorption spectra and FFCF parameters.

	***ν^a^* (cm^−1^)**	**fwhm^b^ (cm^−1^)**	**rel. area^c^ (%)**	Γ^*^(cm^−1^)	**Δ_1_ (cm^−1^)**	**t_1_ (ps)**	**Δ_2_ (cm^−1^)**	**rel. pop.^d^ (%)**
wt	1,939.4 ± 0.2	12.9 ± 0.5	100	1.6	3.0	20.7	4.1	100
L358P	1,937.5 ± 0.3	10.2 ± 0.4	70 ± 6					54
	1,931.2 ± 0.4	8.5 ± 0.3	30 ± 6	3.4	2.6	30.5	2.5	46
Pdx-bound	1,936.8 ± 0.2	8.6 ± 0.3	35 ± 5					25
	1,931.3 ± 0.1	7.6 ± 0.3	65 ± 5	2.2	2.0	31.7	1.8	75

a Center frequency determined from Gaussian fits to linear spectra.

b Full width at half maximum of Gaussian fits to linear spectra.

c Relative integrated absorbance of components in linear spectra.

d *Relative population of states accounting for difference in transition dipole strength*.

**Figure 3:**
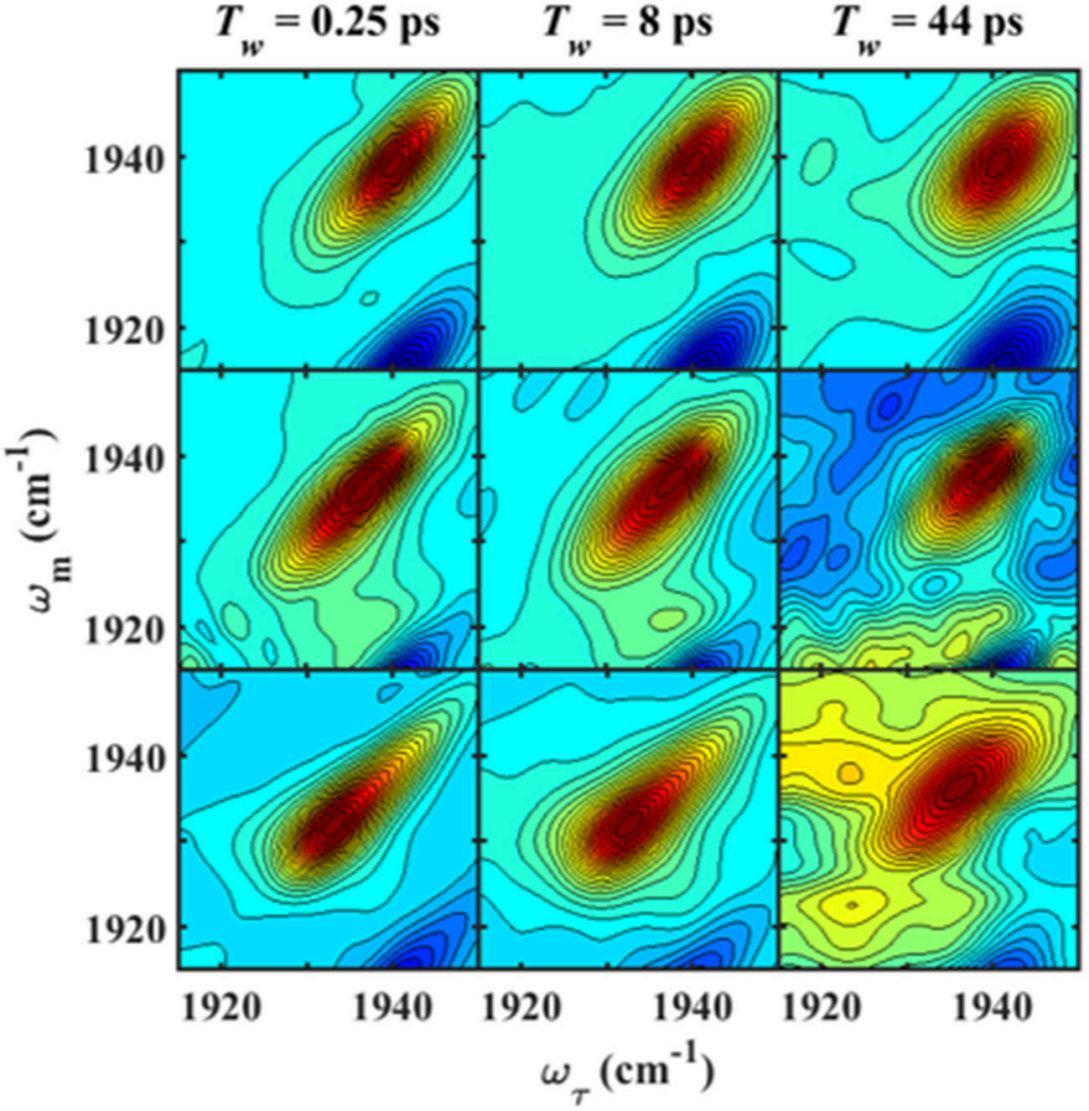
*T*_w_-dependent 2D IR spectra of heme-bound CO for free wt (top row), L358P (middle row), and the Pdx complex (bottom row).

We next characterized the free wt, L358P, and Pdx complex by 2D IR spectroscopy (Figure 3). 2D IR correlation spectra were generated that connect the initial frequencies of the distribution underlying the absorption bands to the frequencies following a time delay, *T*_w_. Analysis of the 2D spectra provided information about the heterogeneity of frequencies underlying the bands (i.e., inhomogeneous broadening), as well as the dynamics of their interconversion. At the shortest *T*_w_ (0.25 ps), the 2D bands were highly elongated along the diagonal (Figure 3, left panels). The spectral elongation reflects high correlation between the initial frequencies (ω_1_ axis) and those after the short *T*_w_ (ω_3_ axis) because the system did not have much time to evolve. As the *T*_w_ delay was lengthened, the proteins had increasing time to interconvert among their distribution of states, leading to different final than initial CO frequencies, so the 2D spectra appeared less elongated due to the loss of frequency correlation (e.g., at *T*_w_ of 44 ps).

**Figure 4:**
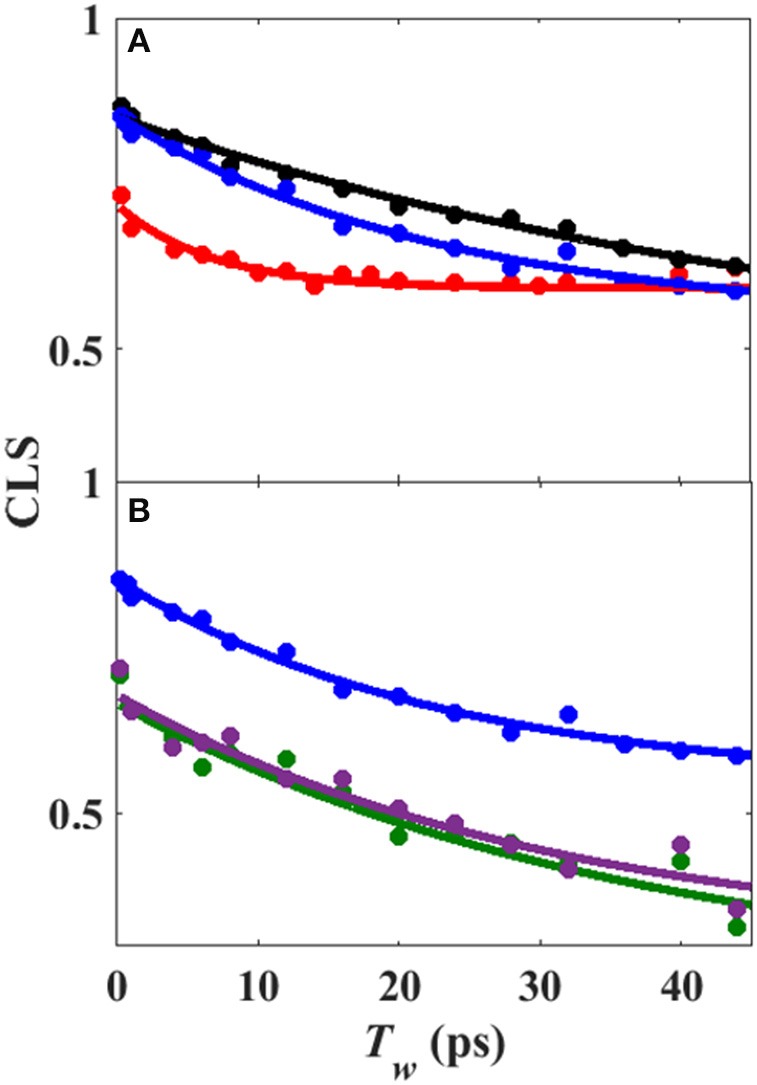
**(A)** CLS decays (points) with fits to exponential decays (lines) from analysis of 2D IR spectra assuming single component for free wt (blue), L358P (black), and Pdx complex (red). **(B)** CLS decays (points) with fits to exponential decays (lines) from analysis of 2D IR spectra in which the CLS decay at higher frequency was assumed equivalent to free wt (black) and that for the component at lower frequency was extracted for L358P (green) and Pdx complex (purple).

Analysis of the *T*_w_-dependent 2D IR spectra for each sample was performed to obtain the CLS decay, which approximates the inhomogeneous part of the normalized FFCF (Kwak et al., 2007), a quantity that reflects the dynamics of the protein environment coupled to the CO oscillator. The initial value of the CLS provides a measure of the inhomogeneous broadening (i.e., the part of the line width due to frequency heterogeneity from population of a distribution of conformational substates), and the decay of the CLS reflects the timescales over which the inhomogeneous distribution is sampled. The CLS decay and the linear spectrum were co-fit to determine the complete FFCF, which includes the homogeneous contribution, approximately those dynamics that are fast on the IR timescale (Δτ≪1), and deconvolutes the inhomogeneous contribution to provide the variance in frequencies (Δ^2^) sampled on a particular timescale (τ). Analysis of the 2D IR data for the free wt enzyme yielded an identical FFCF as determined previously (Table 1; Basom et al., 2015). The FFCF shows three components: fast homogeneous dynamics, inhomogeneous dynamics on a ~20 ps timescale, and inhomogeneous dynamics longer than the experimental timescale (~100 ps). The CLS decays determined for the Pdx complex and L358P by analysis around the maximum of the 2D spectra, assuming a single component, are substantially different from each other and from the free wt (Figure 4A). The initial CLS is significantly smaller for the Pdx complex. The CLS decays for the Pdx complex and L358P also reflect much slower dynamics, indicating very slowly interconverting states.

**Figure 5:**
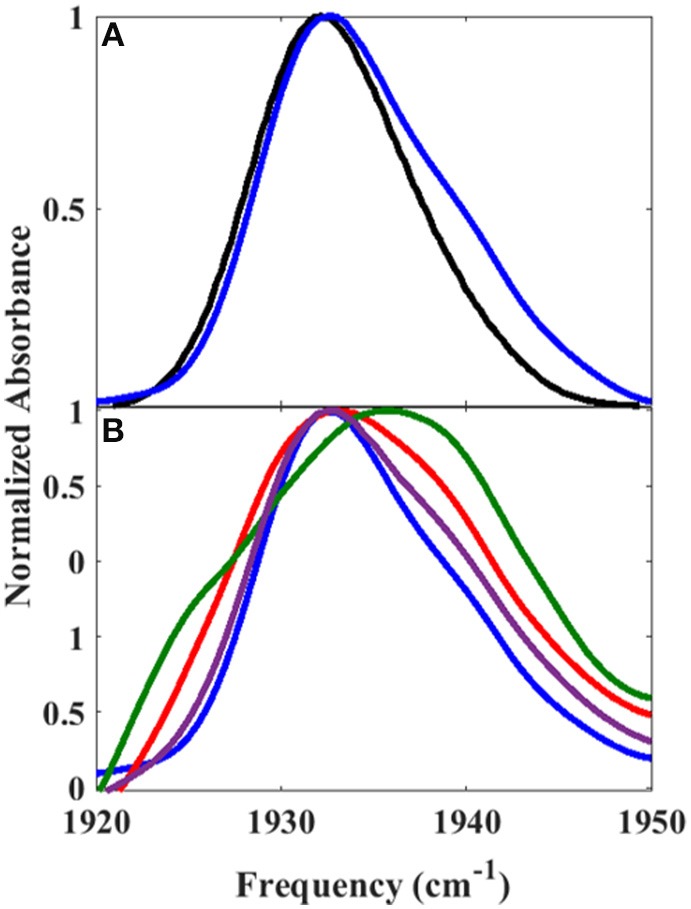
Overlay of **(A)** 2D diagonal slice (*T*_w_ of 0.25 ps) (blue) and FT IR spectrum (black) of Pdx complex and **(B)** 2D diagonal slices at *T*_w_ of 0.25 ps (blue), 20 ps (purple), 32 ps (red), and 44 ps (green).

Since the linear spectra for the Pdx complex and L358P were better fit by two component bands, which suggests the population of two distinct conformational states, a more comprehensive analysis of the 2D data was performed. Indeed, closer inspection of the 2D IR data found clear evidence for two component bands. The 2D IR spectrum at *T*_w_ of 0.25 ps for the Pdx complex shows obvious asymmetry along the diagonal, with the maximum of absorbance substantially lower in frequency than the center of the entire band (Figure 3). The presence of multiple components was even more apparent from overlay of the diagonal slice through the 2D spectrum with the linear FTIR spectrum (Figure 5A, Supplementary Figure 9). The intensity in a 2D spectrum depends on the fourth power of the transition dipole strength of a vibrational transition, whereas that for the linear spectrum depends on the square, such that vibrational transitions with larger transition dipole strengths are accentuated in a 2D spectrum (Hamm and Zanni, 2011). As a result of this difference, the component band at higher frequency that manifested only as slight asymmetry in the linear spectrum appeared as a clear shoulder band in the 2D diagonal slice. Moreover, comparison of the absorption intensity between the linear and 2D spectrum indicated a ~1.3-fold greater transition dipole strength for the CO vibration associated with the band at higher frequency than the one at lower frequency (Supplementary Table 3).

Further evidence for the presence of multiple components in the spectra for the Pdx complex and L358P is provided by the observation of a shift in the first moment of the diagonal 2D slices to higher frequency with increasing *T*_w_ (Figure 5B, Supplementary Table 2). This behavior indicates that the two CO vibrations have distinct vibrational lifetimes in addition to distinct transition dipole strengths. Accordingly, fitting the *T*_w_-dependent amplitude of the diagonal slices yielded lifetimes that are frequency-dependent (Supplementary Figure 8). A global fit to the diagonal spectral data modeling two distinct components gave vibrational lifetimes of 13 ps and 16 ps for the lower and higher frequency bands, respectively. Due to the different lifetimes, the component bands decayed at different rates to result in an apparent shift in the 2D IR spectra to higher frequency with increasing *T*_w_ (Figure 3). The effect has become unmistakable by *T*_w_ of 44 ps, when the lower frequency component has mostly decayed, resulting in the 2D spectrum showing a maximum at the higher frequency component.

**Figure 6:**
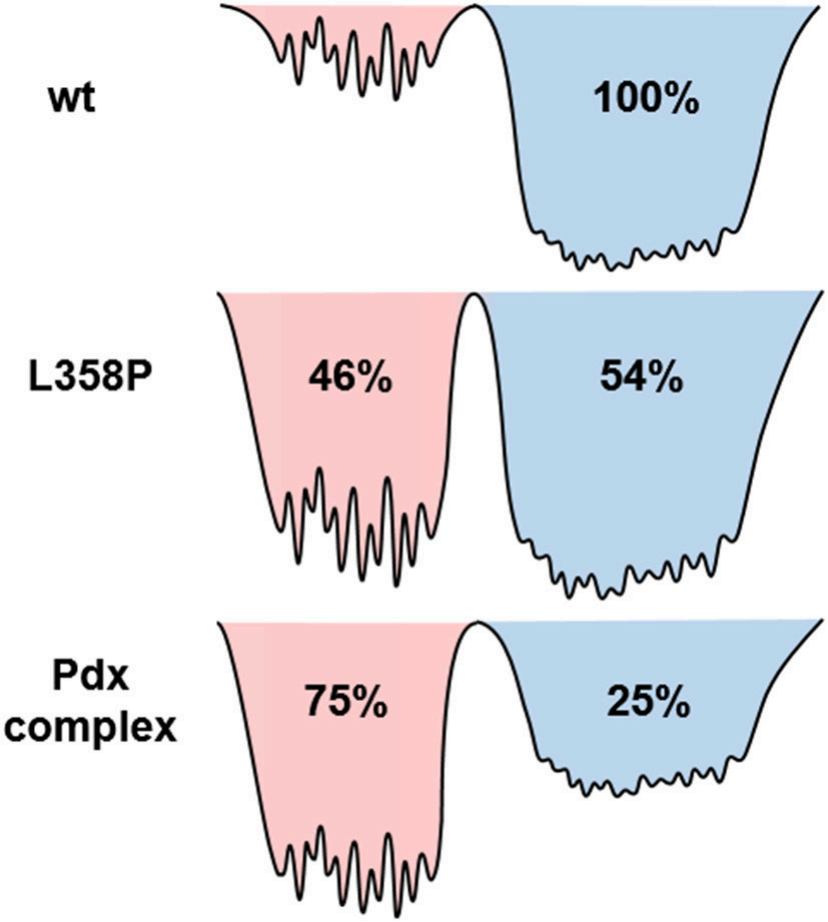
Model illustrating the differences among the energy landscapes of the free wt, L358P, and Pdx complex based on the IR data. The conformational wells shaded in blue and red reflect the states associated with the bands at high and low frequency, respectively. The relative depths of the wells qualitatively reflect the differences in the population of the conformations. The breadth of the well reflects the inhomogeneous broadening in the state, and the height of the barriers among the substates are estimated from the different timescale of the dynamics.

Since the spectra for the Pdx complex and L358P appeared to contain two components, CLS analysis was performed for the *T*_w_-dependent 2D IR data assuming a superposition of two distinct bands, each with a distinct FFCF. The CLS decay for the band at lower frequency was assumed to reflect the FFCF determined for the single absorption band found for the free wt enzyme. With this CLS decay, the relative populations of the two states obtained from the band fitting, and the lifetimes determined from analysis of the time-dependent 2D diagonal spectra, the CLS decay for the second, lower frequency component was extracted from the 2D data for the Pdx complex and L358P. The CLS decays determined for the lower frequency components of the Pdx complex and L358P well-overlapped (Figure 4B), indicating that they reflect the same conformational state with the same energy landscape of substates. Thus, the data were well-modeled by a superposition of the conformation populated by the free wt enzyme and a second state associated with the absorption at lower frequency that is induced by Pdx binding or the L358P mutation. The different FFCFs imply distinct local energy landscapes in the conformations (Figure 6). Compared to the free wt enzyme, the FFCF of the induced state shows that the CO experiences ~30% less inhomogeneous broadening, suggesting lower heterogeneity of states. In addition, the timescale of dynamics measured among inhomogeneous states of 30 ps for the induced state was also slower than the 20 ps measured for the free wt (Table 1). Slower dynamics reflect a more rugged energy landscape, with the 1.5-fold change corresponding to ~4.5 higher energy barriers among substates.

## Discussion

The IR data indicate that the reduced, CO-ligated state of the Pdx complex and L358P populate multiple conformations—the closed state and a second conformation. An earlier report of the linear CO spectra with increasing addition of Pdx noted the asymmetry of the band at high Pdx concentrations, but the feature was attributed to residual unbound P450cam (Nagano et al., 2003). Similarly, the mutation L358P was found to shift the IR absorption of CO to lower frequency (Yoshioka et al., 2000), but the presence of multiple bands was not uncovered. In contrast, the 2D IR data unequivocally report two distinct CO absorptions that provide evidence that the Pdx complex and L358P significantly populate multiple distinct states. That the CO has distinct transition dipole strength in the two states is evident from the different absorption intensity at low and high frequency found for the linear and 2D diagonal spectra (Figure 5A). In addition, the observed shift in the first moment of the 2D spectra with increasing *T*_w_ indicates that the CO ligands in the two states have different vibrational lifetimes (Supplementary Figures 7, 8). As expected in this case, at long *T*_w_ times the 2D spectra become dominated by the component at higher frequency with the longer vibrational lifetime, which otherwise only manifests as slight asymmetry in the linear IR spectra. Furthermore, the FFCFs that reflect the coupled dynamics of the environment are distinct for the bands (Figure 4B; Table 1), implying different protein motions are experienced by the CO within each state.

In agreement with previous studies (Myers et al., 2013; Liou et al., 2016), the IR data indicate that the Pdx complex of ferrous, CO-ligated P450cam populates a state similar to the closed state observed in crystal structures of the free enzyme, although this state appears to be the minor species. The correspondence of this closed state to the higher frequency CO band is strongly supported by the success in modeling the *T*_w_-dependent 2D data for both the Pdx complex and L358P under the assumption that the FFCF was the same as that for the free wt sample, which is accepted to be in the closed state. Because the FFCF reflects the coupled dynamics of the environment, the observation of equivalent FFCFs means that the CO is sensitive to the same protein motion in the state associated with the high frequency band as in the free wt enzyme. Also in agreement with previous studies (Myers et al., 2013; Liou et al., 2016), the data is not consistent with population of the open state. Previous 2D IR investigation of the CO complex of substrate-free P450cam (Thielges et al., 2011), which adopts the open state (Asciutto et al., 2009; Lee et al., 2010), observed CO absorptions at higher frequencies and determined FFCFs that dramatically differ from those obtained for the Pdx complex. Overall, accounting for the difference in transition dipole strengths, we found that 25% population of the Pdx complex and 54% population of L358P adopt the closed state (Supplementary Table 3).

Although the band at higher frequency is reasonably assigned as the closed state, the small variation observed in the frequencies for the Pdx complex and L358P from the free wt enzyme (lower by 1.9 and 2.6 cm^−1^, respectively) indicate some difference among the structures. A probable explanation is perturbation of the proximal side of the heme. The mutation L358P removes an electrostatic interaction of the amide backbone of L358 with the thiolate of the cysteine ligand. As suggested previously (Yoshioka et al., 2000), this interaction is likely to withdraw electron density from the heme iron, and ultimately out of a π^*^ orbital of the CO. Thus, the removal of the amide-thiolate interaction via introduction of L358P is expected to decrease CO bond order and the vibrational frequency. The perturbation however does not appear to affect the FFCF, thus the enzyme motion coupled to the CO vibration.

The question remains what structural change is reflected by the appearance of the second, lower frequency band. One possibility is that the two CO bands are due to co-population of a loosely bound encounter complex and a more tightly associated complex (despite that the P450cam was expected to be >95% bound by Pdx considering the protein concentrations and *K*_D_; Supplementary Material Sample Preparation). Analogous models have been proposed for other redox partners, which in general tend to form dynamic complexes (Skourtis et al., 2010; Bashir et al., 2011; Cruz-Gallardo et al., 2012). A previous study of the Pdx complex employing NMR paramagnetic relaxation enhancement found evidence for the population of a minor state, which was attributed to a tightly bound complex, while the major state was attributed to an encounter complex with P450cam in the closed conformation (Hiruma et al., 2013). In contrast, we find that the 25% populated state, which is associated with the band that exhibits spectral features more similar to the free enzyme, likely corresponds to the closed conformation in the encounter complex. The lower frequency band then is assigned to 75% population of a second, possibly more tightly bound, complex of P450cam and Pdx. In addition, our data indicate that the introduction of the mutation L358P promotes this second state even in the absence of Pdx, leading to its population about equal to the closed state. This interpretation is in line with previous observations that L358P induces spectral perturbations that are similar, but not identical, to those resulting from Pdx binding (Yoshioka et al., 2000, 2002; Tosha et al., 2004).

**Figure 7:**
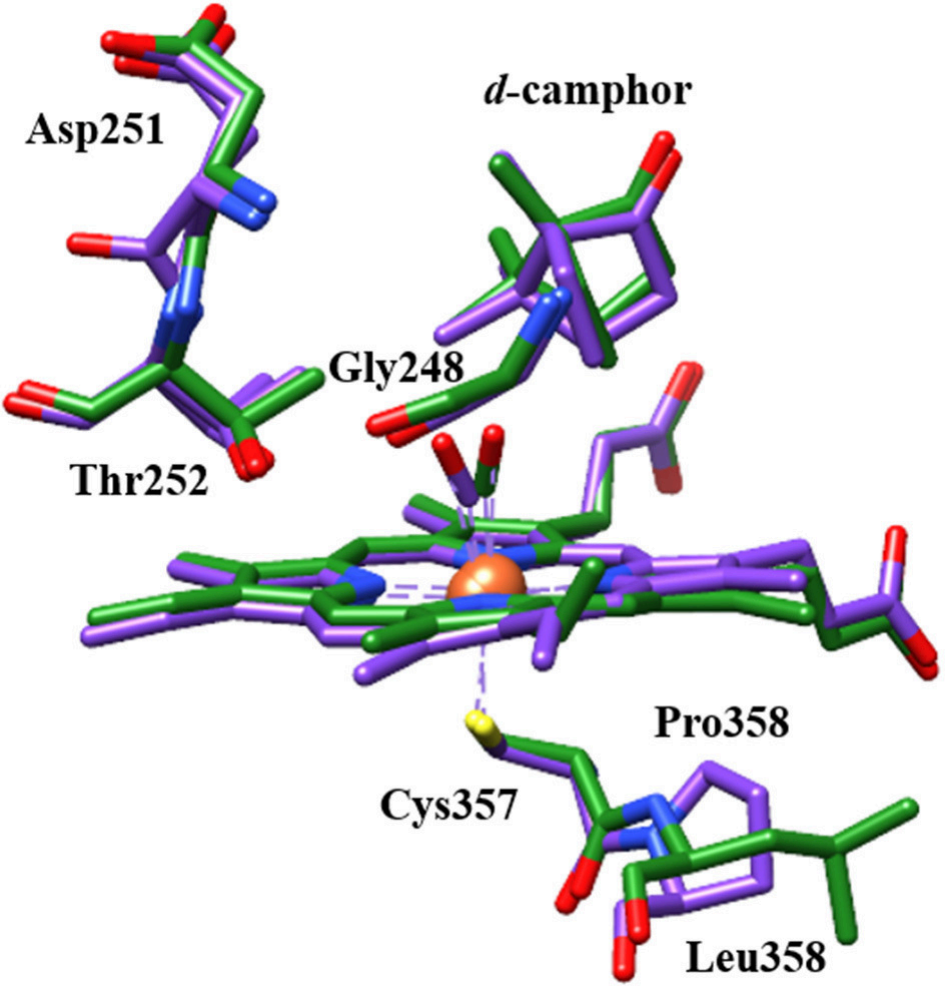
Structural comparison of CO-ligated wt P450cam (green) and L358P (purple) showing CO, heme, camphor substrate, and several residues of the proximal and distal pockets. Protein crystal structures (PDB ID: 1T87 and 1T85) were superimposed using UCSF Chimera (Pettersen et al., 2004).

A potential structural basis for the low frequency band is suggested by the crystal structure of L358P (Nagano et al., 2004). In both wt and L358P structures, the CO ligand is directed toward a groove in helix I, where disruption in hydrogen bonding occurs at Gly248 due to its amide oxygen alternately interacting with the side chain of residue Thr252 (Figure 7; Raag and Poulos, 1989; Nagano et al., 2004). Compared to wt, the L358P structure shows movement of the terminal oxygen of the CO ligand closer to the side chain of Thr252 along with slight displacement of the camphor substrate. These changes are similar to, but less dramatic than, those observed for the O_2_ complex, where the hydrogen bond between Thr252 and the backbone of Gly248 is broken, and one forms between Thr252 and the O_2_ ligand (Schlichting et al., 2000). The groove in the helix I also widens, and two water molecules enter the pocket. Such changes are thought to facilitate the proton relay network required for O-O bond scission following the second reduction. The possibility that a similar conformational change occurs for CO-ligated P450cam upon Pdx binding is supported by NMR studies, which propose a connection between the changes in the active site to a cis/trans isomerization of Pro89 at a distant location (Asciutto et al., 2009).

Analogous structural changes for the L358P or Pdx complex are likely to result in substantial perturbation to the CO frequency. In particular, the lower frequency observed for L358P and the Pdx complex is consistent with the expected influence of a hydrogen bonding interaction between CO and Thr252 (Spiro and Wasbotten, 2005). Another possibility is supported by the observation from crystallographic study of L358P of electron density for a number of residues that indicate population of multiple conformations (Nagano et al., 2004). Thr252 appears to adopt a second conformation in which side chain rotation places the methyl group instead of the hydroxyl group closer to the CO ligand. The weaker interaction of the methyl group than the polar hydroxyl group with the CO vibration is likely to engender less inhomogeneous broadening, which is consistent with the FFCF determined for the lower frequency component. Further work to model the spectral data using molecular dynamics simulations should enable better understanding of the structural origins.

Significantly, this study provides insight into the perturbation to the conformational ensemble induced by Pdx binding to ferrous, CO-ligated P450cam. We observed that the Pdx complex populates two states that potentially reflect a loosely bound encounter state and a more tightly bound state, in line with our growing understanding of the highly dynamic complexes formed by electron transfer partners (Skourtis et al., 2010; Bashir et al., 2011; Cruz-Gallardo et al., 2012). In comparison to the O_2_ complex, the ferrous, CO complex does not bear a partial negative charge on the terminal oxygen of the ligand, but nevertheless reflects a one-electron reduced state with a bound diatomic ligand. The O_2_ complex is generally considered a ferric peroxo species but is likely in equilibrium with the ferrous dioxygen species (Denisov et al., 2005). In further studies, 2D IR spectroscopy of a CN^−^ ligand as an IR probe could provide information about the conformational ensemble at the same location in P450cam as CO, but enable comparison to a ferric ligand complex. As in our previous study of camphor binding to P450cam (Basom et al., 2016), incorporation of IR probe groups at amino acid side chains will permit characterization of the effect of Pdx binding at locations in P450cam beyond the distal heme pocket, and potentially enable study of the O_2_ complex itself. Nevertheless, the spectral features of the state induced by Pdx binding in this study of the CO-ligated P450cam are consistent with conformational changes that would facilitate subsequent steps in the catalytic cycle found in structural studies (Poulos, 2005), supporting the involvement of the induced state detected in this study in the effector role of Pdx.

## Data availability statement

The raw data supporting the conclusions of this manuscript will be made available by the authors, without undue reservation, to any qualified researcher upon request.

## Author contributions

SR performed spectroscopic experiments and sample preparation, analyzed the data, and wrote the paper; EB performed protein expression and purification and edited the paper; MT designed the research, and wrote the paper.

### Conflict of interest statement

The authors declare that the research was conducted in the absence of any commercial or financial relationships that could be construed as a potential conflict of interest.
