# Supplementary material for: Conformational Change Induced by Putidaredoxin Binding to Ferrous CO-ligated Cytochrome P450cam Characterized by 2D IR Spectroscopy
Source: Front Mol Biosci. 2018 Nov 13;5:94. doi: 10.3389/fmolb.2018.00094 (PMC6243089; doi:10.3389/fmolb.2018.00094)
Supplement: Supplementary file 1 [file Data_Sheet_1.pdf]

Supplementary Material

Conformational Change Induced by Putidaredoxin Binding to Ferrous CO-ligated P450cam Characterized by 2D IR Spectroscopy

Sashary Ramos, Edward J. Basom, Megan C. Thielges*

*** Correspondence:** Corresponding Author: thielges@indiana.edu

1 Experimental Methods

1.1 Expression and Purification of Protein

Plasmids (pDNC334A and pKM36) for the expression of cytochrome P450cam (P450cam) and putidaredoxin (Pdx), as well as cell strain NCM533 were kindly provided by Thomas Pochapsky (Brandeis University) (Lyons et al., 1996; OuYang et al., 2006). The wild-type (wt) sequence contains the mutation C334A that reduces protein aggregation, but has been shown not to affect the enzymatic activity (Nickerson and Wong, 1997). L358P was introduced into the P450cam sequence using standard site-directed mutagenesis (Stratagene, Agilent) and confirmed by sequencing.

P450cam variants were expressed and purified as previously described with some modifications (Basom et al., 2015). The wt and L358P were expressed in BL21(DE3) in Luria Broth in the presence of 33 mg/L chloramphenicol at 37 °C with 250 rpm shaking for 14 hours. Expression was induced at OD_{600} = 0.6 by IPTG, at which point the cultures were brought to 0.5 mM δ-aminolevulinic acid and 0.5 mM *d*-camphor. The cells were harvested by centrifugation and resuspended in 50 mM potassium phosphate, pH 7.0, 100 mM KCl, 1 mM *d*-camphor, 1 mM EDTA. The cells were then lysed by sonication, treated with DNase-1 (New England Biolabs) and centrifuged at 15,000 rpm at 4 °C for 30 min. The supernatant was dialyzed into 20 mM potassium phosphate, pH 7.0, 400 mM KCl, 1 mM *d*-camphor, 1 mM EDTA, filtered and passed over a Q column (Bio-Rad). The eluate was then dialyzed into 20 mM potassium phosphate, pH 7.0, 1 mM *d*-camphor and passed over a second Q column that was equilibrated with 20 mM potassium phosphate, pH 7.0. The protein sample was eluted with a linear KCl gradient from 0 – 400 mM. Fractions with $A_{390/280} \geq 0.7$ were combined and further purified with a S100HR Sephacryl column (GE Life Sciences) in 50 mM potassium phosphate, pH 7.0, 100 mM KCl, 1 mM *d*-camphor. Fractions with $A_{390/280} \geq 1.3$ were combined, brought to 50% glycerol (w/v), flash frozen in liquid N_2, and stored at -80 °C.

All buffers used in the expression and purification of Pdx were degassed and purged with $Ar_{(g)}$. Pdx expression and purification was performed as previously described with modifications (Liou et al., 2016). The plasmid pKM36 was transformed into NCM533 and grown in Luria Broth with 100 μg/mL ampicillin and 30 μg/mL kanamycin. Starter cultures were used to inoculate 1 L expression cultures in Terrific Broth containing 100 μg/mL ampicillin and 30 μg/mL kanamycin. Expression was induced at $OD_{600} \approx 1.5$ by IPTG and proceeded for 24 hours at 37 °C with shaking at 250 rpm. Cells were harvested by centrifugation and pellets were flash frozen and stored at -80 °C until purification. The cells were then resuspended in 50 mM Tris·Cl, pH 8.0 and treated with DNase-1, RNase, lysozyme and tosyl chloride. The cell resuspension was stirred for 1 hour then lysed by sonication. The protein

was unfolded by treatment of the lysate with 8 M urea and stirring under $Ar_{(g)}$ for 30 min. The solution was brought to 10 mM in DTT and stirred for an additional 5 min. under $Ar_{(g)}$. $FeCl_2$, and Na_2S then were added to 1 mM. After 10 min. of additional stirring under $Ar_{(g)}$, the solution was diluted 3-fold with degassed 50 mM Tris·Cl, pH 8.0 to induce refolding, stirred for 10 min. under $Ar_{(g)}$, diluted ~2-fold by non-degassed buffer, and finally stirred for another 10 min. in open air. The solution was centrifuged, and the supernatant was passed through a 0.2 µm filter and loaded onto a DEAE column (Bio-Rad) equilibrated with 50 mM Tris·Cl, pH 8.0, 0.1 mM DTT. The protein was eluted with a linear gradient of 0 – 400 mM KCl. To increase purity, the protein was then precipitated by addition of $(NH_4)_2SO_4$ to 85% saturation and centrifuged. The recovered pellet was resuspended in 50 mM Tris·Cl, pH 8.0 with 100 mM KCl, concentrated by centrifugation and run over a Bio-Gel P-30 (Bio-Rad) column equilibrated with 50 mM Tris·Cl pH 7.4. Fractions with $A_{325/278} \geq 0.65$ were combined, brought to 50% glycerol (w/v), flash frozen in liquid N_2, and stored at -80 °C.

1.2 Sample Preparation

P450cam and Pdx samples were dialyzed into 100 mM potassium phosphate, pH 7, 50 mM KCl, 5 mM *d*-camphor and concentrated by spin concentration. Protein concentration was determined by UV-visible absorbance spectroscopy; an extinction coefficient of ε_{390} = 100 $mM^{-1}cm^{-1}$ was used for camphor-bound P450cam and ε_{415} = 11.1 $mM^{-1}cm^{-1}$ and ε_{455} = 10.4 $mM^{-1}cm^{-1}$ were used for Pdx (Peterson, 1971; Gunsalus and Wagner, 1978). In all cases, the concentration of camphor was such that P450cam would be >99% bound. Pdx samples were reduced by 10 equivalents of DTT and stored under $Ar_{(g)}$. To prepare the wt and L358P P450cam-carbon monoxide complex, 10 µL aliquots of the concentrated protein samples were placed in tubes and sealed with rubber septa. The headspace of each sample was purged under $Ar_{(g)}$ for 10 min. and the protein was reduced with 15 equivalents of sodium dithionite. The headspace was then purged with CO for 10 min., agitating gently, in order to bind CO. For the P450cam-putidaredoxin complex, reduced Pdx was added to the reduced P450cam before purging with CO. The samples were prepared to contain P450cam >95% bound by Pdx, on the basis of a K_D of 24 µM (Nagano et al., 2003; Pochapsky et al., 2003) and final protein concentrations of 1 mM P450cam and 1.6 mM Pdx. CO-bound samples were immediately loaded between two 2 mm CaF_2 windows with a 76.1 µm Teflon spacer. The visible spectra of each sample were checked before and after data collection. The signature peak at ~446 nm for the CO-ligated camphor complex of P450cam was observed for wt, L358P and the Pdx complex (Supplementary Figure 1).

The FT IR spectra of each sample was collected before 2D IR experiments at 2 cm^{-1} resolution with an Agilent Cary 670 FT IR spectrometer equipped with a liquid N_2-cooled MCT detector. All spectra were collected after a 20-minute purge with dry $N_{2(g)}$. All samples were loaded between two 2 mm thick CaF_2 windows and separated by a 76.1 um Teflon spacer. Sample and background spectra were averaged over 2,500 scans and all interferograms were processed using a 4-term Blackman-Harris apodization function and a zero-filling factor of 8. Background spectra were collected of the protein complexes sans CO in 100 mM potassium phosphate, pH 7, 50 mM KCl, 5 mM *d*-camphor. Slowly varying baselines were removed by fitting and subtracting a polynomial from the spectral regions excluding the CO band.

The CO spectra for each sample was fit to on or a sum of two Gaussian functions. The spectra for the Pdx complex and L358P were best fit by sum of two Gaussian functions while that for the free wt was adequately modeled by one. Comparisons of the one and two component fits to the spectra for the Pdx complex and L358P are shown in Supplementary Figure 2. The first spectral moment for the Pdx complex is not the same as the frequency of maximum absorbance (Supplementary Table 1),

suggesting that multiple components underlie the spectrum. A similar comparison for L358P does not immediately indicate a second component; however, the second derivative spectrum suggests a possible second component (Supplementary Figure 3). In addition, F-tests at a 95% confidence level indicate that inclusion of a second Gaussian component significantly improves the fits to the spectra for the Pdx complex and L358P (Supplementary Table 1).

1.3 2D IR and Pump Probe Spectroscopy

A Ti:Sapphire oscillator/regenerative amplifier (Spectra Physics) producing ~75 fs pulses centered at 800 nm with 1 kHz repetition rate was used to pump a home-built optical parametric amplifier (OPA) to produce tunable mid-IR pulses. The optical parametric amplifier was tuned to produce ~20 μJ, ~150 fs mid-IR pulses centered at 1920 cm^{-1} (90 cm^{-1} FWHM), which encompassed both the 0-1 and 1-2 vibrational transitions of the CO probe. Linear chirp was measured via FROG and corrected to less than 0.02 fs/cm^{-1} with insertion of CaF_2 or Ge substrates in the beam path. The described system was used for both 2D IR and pump probe spectroscopy experiments.

Pump probe experiments were conducted as previously described (Le Sueur et al., 2017; Ramos et al., 2017). Briefly, the mid-IR beam was split into a pump and probe beam, focused to ~300 μm (99% diameter) and spatially overlapped at the sample. The delay between the pump and probe pulses was varied using a computer-controlled delay stage (Aerotech) and pulse temporal overlap was set using the two-photon pump-probe signal observed from an indium arsenide wafer. The pump beam was chopped at 500 Hz and the probe beam was frequency-dispersed onto a 32-pixel liquid N_2-cooled MCT detector array (Infrared Associates). The pump-induced change in the transmission of the probe beam was measured as a function of pump-probe delay. Background signals were corrected for by the subtraction of the difference spectrum for the longest pump-probe delay (200 ps), when the contribution from the CO was assumed to be negligible. The pump-probe spectra at each frequency were then fit to a single exponential decay (Supplementary Figure 4).

2D IR vibrational echo spectra were collected as previously reported with some modifications (Park et al., 2007; Basom et al., 2015; Kramer et al., 2016). The mid-IR was split along four separate paths, beam 1, 2, 3 and the local oscillator (LO). Beams 1, 2 and the LO were temporally controlled using computer-controlled delay stages (Aerotech). The timing between the pulses of beam 1, 2 and 3 was determined by a cross-correlation measurement with the non-resonant signal produced by CCl_4. A reference beam was split from the LO and detected on a single-element liquid N_2-cooled MCT detector to account for shot-to-shot laser fluctuations. In a 2D IR experiment, the application of three pulses (beams 1, 2 and 3) onto the sample lead to emission of a third order signal in the phase-matched ($-k_1 + k_2 + k_3$) direction. The third-order signal is heterodyne-detected by overlapping with the LO to provide amplification and phase information. The combined signal and LO are dispersed by a spectrograph (Horiba iHR320, 300 grooves/mm grating) onto a 32-pixel liquid N_2-cooled MCT detector. A single 2D spectrum is generated by scanning the time between the first two pulses (τ) while the time period between the second and third pulses (T_w) is held constant. The heterodyned third-order signal generated after the application of the third pulse produces an interferogram along τ, which is Fourier transformed to produce the ω_1 axis. Frequency-resolved detection with the MCT array generates the ω_3 axis of the 2D spectrum. All 2D IR spectra were phase-corrected as previously described (Park et al., 2007).

Quasi-rotating frame (QRF) 2D IR experiments were conducted as previously reported (Kramer et al., 2016). QRF experiments allow for faster acquisition of data by shifting nonlinear signals into a rotating

frame in τ to decrease the effective oscillation frequency, thus decreasing the sampling necessary to achieve the Nyquist criterium. Experimentally, this causes the LO to be delayed to a fraction, f, of τ, later than pulse 3 for positive τ values and ahead of pulse 3 for negative τ. To shift QRF data to the stationary frame, an array calibration interferogram is generated by scanning the LO time delay through a range longer than f times $-\tau$ to τ. This calibration interferogram is then used to determine the correct frequencies with which to shift the ω_1 axis to obtain the stationary frame. For the experiments for free wt and L358P, a QRF fraction, f, of 0.848 was used and an array calibration interferogram was collected by scanning the LO decay from -20 to 20 ps with 4 fs steps. 2D IR data of free wt was collected in both stationary frame and quasi-rotating frame, and the CLS curves obtained from each method were identical within error as shown in Supplementary Figure 5.

Slices along the diagonal of the 2D spectra were further analyzed (Supplementary Figure 6, Figure 4B). The first moments of the diagonal slices at increasing T_w for the Pdx complex and L358P were observed to shift to toward the higher frequency component observed in the FT IR spectra (Supplementary Figure 7, Supplementary Table 2). The decays of the 2D diagonal spectra were fit to an exponential function and a frequency-dependent change in lifetime was observed (Supplementary Figure 8). Gaussian fitting of the diagonal slice extracted from a 0.25 ps spectrum of Pdx gave relative integrated absorbance of 46% associated with a lower frequency component and 53% associated with a higher frequency component. These values were compared to the fits of the linear spectra and when taking the transition dipole into account determined relative populations of 25% of the higher frequency component and 75% of a lower frequency component (Supplementary Figure 9, Supplementary Table 3).

1.4 Two-component CLS Determination

Due to the multiple bands exhibited by the Pdx complex and L358P additional CLS analysis of the 2D spectra to account for two components using a previously established method was conducted (Fenn and Fayer, 2011). This method can extract a second component of the FFCF when a first component and the fractional contribution of the component bands are known. The CLS decay obtained from the 2D IR spectra of the wt P450cam was used to the model the contribution of the high frequency component band, and CLS decay of the low frequency component band was determined. The center line data were obtained from the 2D spectra of the Pdx complex and L358P and fit to the following equation:

$$\omega^*_{m,l}(\omega_m, \omega_\tau, T_w) = \frac{\omega^*_{m,2C}(\omega_m, \omega_\tau, T_w) - f_h(\omega_\tau, T_w)\omega^*_{m,h}(\omega_m, \omega_\tau, T_w)}{1 - f_h(\omega_\tau, T_w)}$$

where $\omega^*_{\mathrm{m,l}}$ are the center line data of the unknown low-frequency component, $\omega^*_{\mathrm{m,2C}}$ are the experimentally determined center line data from the two-component spectra of the Pdx complex or L358P, $\omega^*_{\mathrm{m,h}}$ are the center line data of the known high-frequency component (modeled from data for the wt P450cam), and f_h is the weighted contribution of the known high-frequency component at every frequency (ω_τ) as a function of T_w. The parameter f_h depends on the fractional population of the two bands (S_h and S_l), determined from the linear and 2D spectra, and the vibrational lifetime of the high and low frequency component ($T_{1,h}$ and $T_{1,l}$) was set to 16 ps and 13 ps, respectively, for the Pdx complex, and 19 ps and 16 ps for the L358P.

$$f_h(\omega_\tau, T_w) = \frac{S_1(\omega_\tau)e^{-T_w/T_{1,h}}}{S_1(\omega_\tau)e^{-\frac{T_w}{T_{1,h}}} + S_2(\omega_\tau)e^{-T_w/T_{1,l}}}$$

2 Supplementary Figures and Tables

2.1 Supplementary Figures

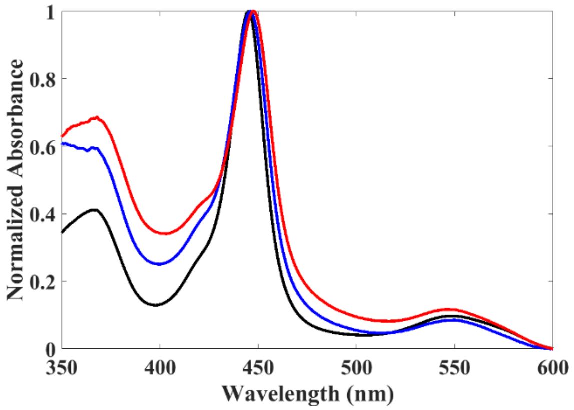

Supplementary Figure 1. Visible absorbance spectra of CO-ligated free wt (blue), L358P (black) and the Pdx complex (red).

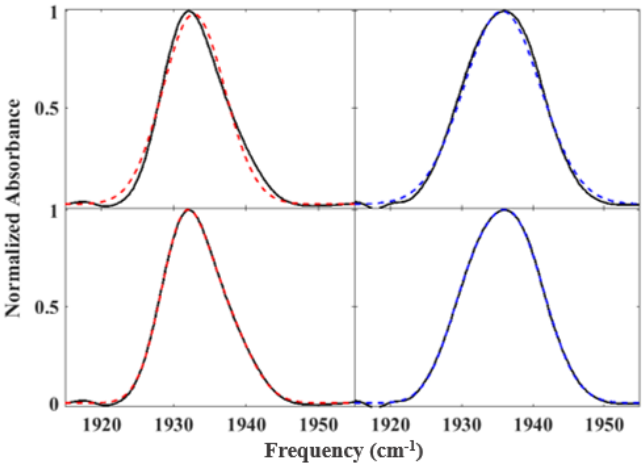

Supplementary Figure 2. Overlay of experimental linear spectra (black solid lines) and fits (dashed lines) including one (upper panels) or two (lower panels) Gaussian components for the Pdx complex (left panels, red) and L358P (right panels, blue).

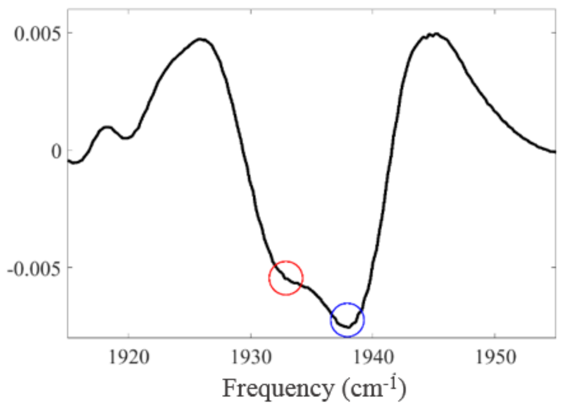

Supplementary Figure 3. Second derivative spectra for L358P indicating multiple components (minima circled in red and blue).

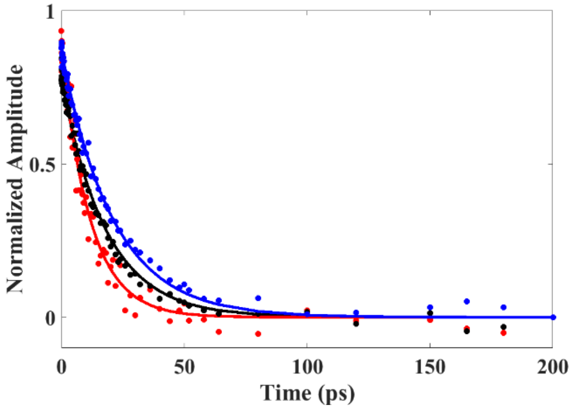

Supplementary Figure 4. Pump probe decays at frequency of maximum absorbance (dots) and exponential fits (lines) for free wt (blue), L358P (black) and the Pdx complex (red).

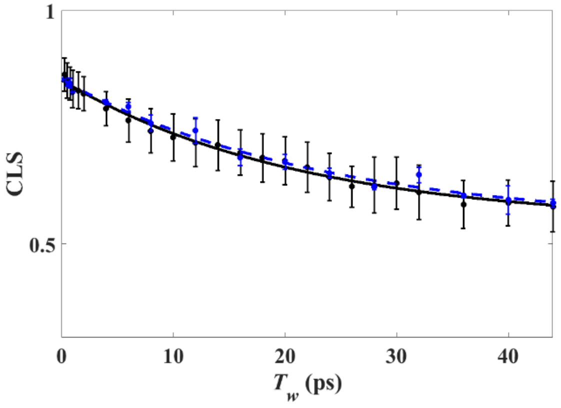

Supplementary Figure 5. CLS decay curves (dots) and fits (lines) collected in stationary frame (black) and quasi-rotating frame (blue) for free wt P450cam.

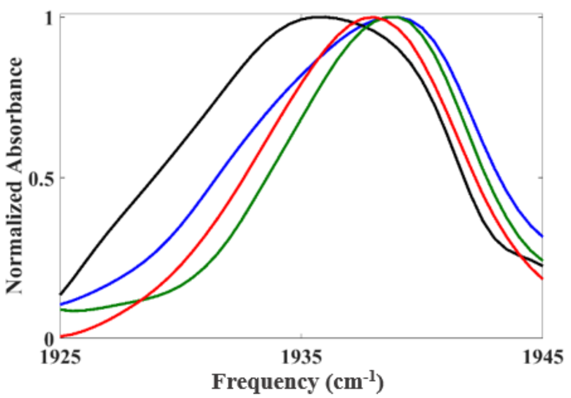

Supplementary Figure 6. Diagonal slices from the 2D spectra of L358P at T_w of 0.25 ps (black), 20 ps (blue), 32 ps (red) and 44 ps (green).

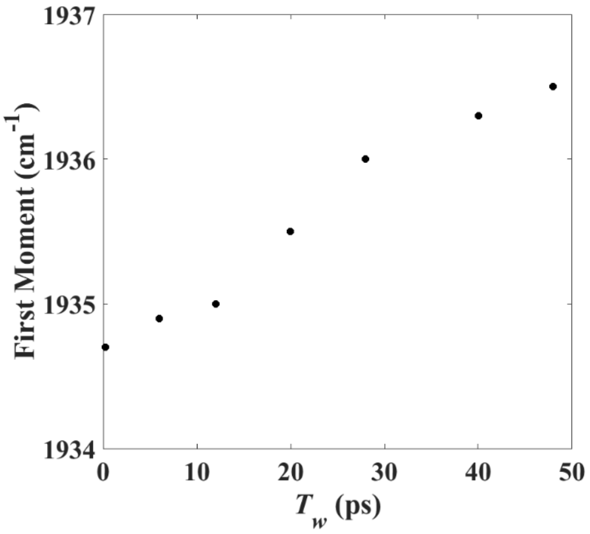

Supplementary Figure 7. T_w–dependence of first moment of 2D diagonal spectra for the Pdx complex.

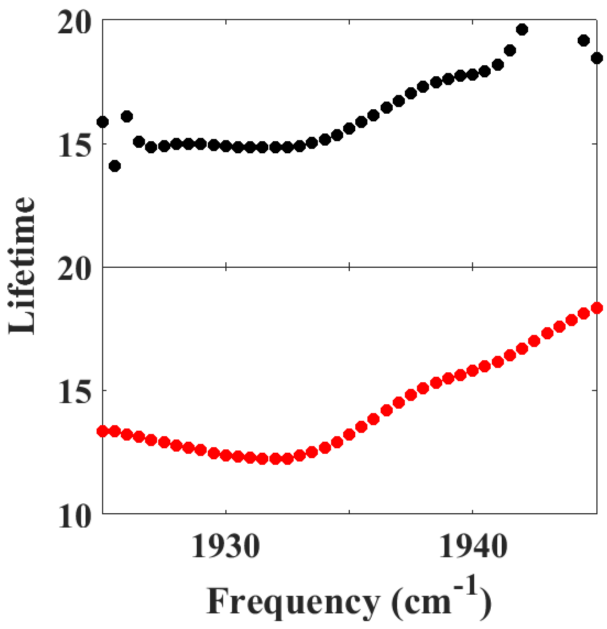

Supplementary Figure 8. Plot of vibrational lifetimes determined as function of frequency from T_w-dependence of 2D diagonal spectra for L358P (upper panel, black) and the Pdx complex (lower panel, red).

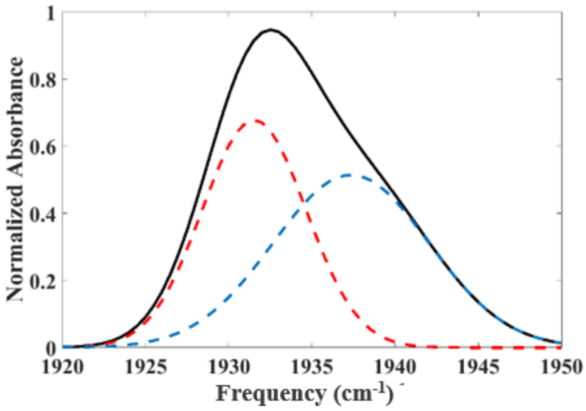

Supplementary Figure 9. Gaussian fits (dashed lines) of a 2D diagonal spectra (solid line) of the Pdx complex collected at T_w of 0.25 ps.

2.2 Supplementary Tables

Supporting Table 1: Parameters describing the linear spectra and band fitting.

	**Max ν (cm^{-1})**	**1st moment**	$\boldsymbol{F_{statistic}}$	$\boldsymbol{^{a}F_{critical}}$
L358P set 1	1936.2	1935.9	16.3	8.8
L358P set 2	1935.9	1935.6	57.2	8.8
L358P set 3	1935.9	1935.5	39.8	8.8
Pdx set 1	1932.1	1933.2	46.8	8.9
Pdx set 2	1932.1	1933.1	47.0	8.9
Pdx set 3	1932.1	1933.4	33.1	8.9

$^{a}F_{critical}$ was calculated at 95% confidence for degrees of freedom of 10 and 13 for L358P and 11 and 8 for the Pdx complex.

Supplementary Table 2: First moments of 2D diagonal slices for Pdx complex.

$\boldsymbol{T_w}$ **(ps)**	**1st moment**
0.25	1934.7
6	1934.9
12	1935.0
20	1935.5
28	1936.0
40	1936.3
48	1936.5

Supplementary Table 3: Relative integrated absorbance and populations of two components for Pdx complex.

**^{a}linear (%)**	**2D (%)**	**transition dipole strength**	**rel. pop. %**
65	47	1.3	75
35	53		25

^{a} Relative integrated absorbance calculated from fits of FT IR spectra to sum of two Gaussian functions.
^{b} Relative integrated absorbance calculated from fits of 2D diagonal extracted at 0.25 ps to sum of two Gaussian functions.
^{c} Relative population of two states accounting for difference in transition dipole strength.

3 References

Basom, E.J., Spearman, J.W., and Thielges, M.C. (2015). Conformational landscape and the selectivity of cytochrome P450cam. *J. Phys. Chem. B* 119**,** 6620-6627. doi: 10.1021/acs.jpcb.5b03896.

Fenn, E.E., and Fayer, M.D. (2011). Extracting 2D IR frequency-frequency correlation functions from two component systems. *J. Chem. Phys.* 135**,** 074502. doi: 10.1063/1.3625278.

Gunsalus, I.C., and Wagner, G.C. (1978). Bacterial P-450cam methylene monooxygenase components: Cytochrome m, putidaredoxin and putidaredoxin reductase. *Methods Enzymol.* 52**,** 166-188. doi: 10.1016/S0076-6879(78)52019-3.

Kramer, P.L., Giammanco, C.H., Tamimi, A., Hoffman, D.J., Sokolowsky, K.P., and Fayer, M.D. (2016). Quasi-rotating frame: accurate line shape determination with increased efficiency in noncollinear 2D optical spectroscopy. *J. Opt. Soc. Am. B* 33**,** 1143-1156. doi: 10.1364/josab.33.001143.

Le Sueur, A.L., Ramos, S., Ellefsen, J.D., Cook, S.P., and Thielges, M.C. (2017). Evaluation of *p*-(^{13}C,^{15}N-cyano)phenylalanine as an extended timescale 2D IR probe of proteins. *Anal. Chem.* 89**,** 5254-5260. doi: 10.1021/acs.analchem.6b04650.

Liou, S.H., Mahomed, M., Lee, Y.T., and Goodin, D.B. (2016). Effector roles of putidaredoxin on cytochrome P450cam conformational states. *J. Am. Chem. Soc.* 138**,** 10163-10172. doi: 10.1021/jacs.6b04110.

Lyons, T.A., Ratnaswamy, G., and Pochapsky, T.C. (1996). Redox-dependent dynamics of putidaredoxin characterized by amide proton exchange. *Protein Sci.* 5**,** 627-639. doi: 10.1002/pro.5560050407.

Nagano, S., Shimada, H., Tarumi, A., Hishiki, T., Kimata-Ariga, Y., Egawa, T., et al. (2003). Infrared spectroscopic and mutational studies on putidaredoxin-induced conformational changes in ferrous CO-P450cam. *Biochemistry* 42**,** 14507-14514. doi: 10.1021/bi035410p.

Nickerson, D.P., and Wong, L.-L. (1997). The dimerization of *Psuedomonas putida* cytochrome P450cam: practical consequences and engineering of a monomeric enzyme. *Protein Eng.* 10**,** 1357-1361. doi: 10.1093/protein/10.12.1357.

OuYang, B., Pochapsky, S.S., Pagani, G.M., and Pochapsky, T.C. (2006). Specific effects of potassium ion binding on wild-type and L358P cytochrome P450cam. *Biochemistry* 45**,** 14379-14388. doi: 10.1021/bi0617355.

Park, S., Kwak, K., and Fayer, M.D. (2007). Ultrafast 2D-IR vibrational echo spectroscopy: a probe of molecular dynamics. *Laser Phys. Lett.* 4**,** 704-718. doi: 10.1002/lapl.200710046.

Peterson, J.A. (1971). Camphor binding by *Pseudomonas putida* cytochrome P-450. *Arch. Biochem. Biophys.* 144**,** 678-693. doi: 10.1016/0003-9861(71)90375-4.

Pochapsky, S.S., Pochapsky, T.C., and Wei, J.W. (2003). A model for effector activity in a highly specific biological electron transfer complex: The cytochrome P450cam-putidaredoxin couple. *Biochemistry* 42**,** 5649-5656. doi: 10.1021/bi034263s.

Ramos, S., Scott, K.J., Horness, R.E., Le Sueur, A.L., and Thielges, M.C. (2017). Extended timescale 2D IR probes of proteins: *p*-cyanoselenophenylalanine. *Phys. Chem. Chem. Phys.* 19**,** 10081-10086. doi: 10.1039/c7cp00403f.
